# Supplementary material for: Phosphatidic acid drives spatiotemporal distribution of Pex30 at ER-LD contact sites
Source: J Cell Biol. 2025 May 23;224(7):e202405162. doi: 10.1083/jcb.202405162 (PMC12101077; doi:10.1083/jcb.202405162)
Supplement: Table S1 — shows the yeast strain list. [file jcb_202405162_tables1.docx]

**Table 1: Yeast strain list**

| **Strain number** | **Name** | **Genotype** | **Source** |
| --- | --- | --- | --- |
| AJY1000 | BY4741 | *MATa his3Δ1 leu2Δ0 met15Δ0 ura3Δ0* | Dharmacon Inc |
| AJY1071 | *sei1Δ* | BY474? *MAT? his3Δ1 leu2Δ0 met15Δ0 ura3Δ0 sei1::KanMx6* | Laboratory collection |
| AJY1072 | *pex30Δ* | BY474? *MAT? his3Δ1 leu2Δ0 met15Δ0 ura3Δ0 pex30::KanMx6* | Laboratory collection |
| AJY1069 | *sei1pex30Δ* | BY474? *MAT? his3Δ1 leu2Δ0 met15Δ0 ura3Δ0 sei1::KanMx6 pex30::KanMx6* | Laboratory collection |
| AJY369 | WT-*PEX30*-2xmCherry | BY474? *MAT? his3Δ1 leu2Δ0 met15Δ0 ura3Δ0 PEX30*-2xmCherry-*URA3* | Laboratory collection |
| AJY1225 | WT-*PEX30*-2xmCherry-*RTN1*-GFP | BY474? *MAT? his3Δ1 leu2Δ0 met15Δ0 ura3Δ0 PEX30*-2xmCherry-*URA3 RTN1-*GFP-*HIS3* | This study |
| AJY1226 | *sei1Δ*-*PEX30*-2xmCherry-*RTN1*-GFP | BY474? *MAT? his3Δ1 leu2Δ0 met15Δ0 ura3Δ0 sei1::KanMx6 PEX30*-2xmCherry-*URA3 RTN1-*GFP-*HIS3* | This study |
| AJY1011 | *sei1Δ*-*PEX30*-2xmCherry | BY474? *MAT? his3Δ1 leu2Δ0 met15Δ0 ura3Δ0 sei1::KanMx6 PEX30*-2xmCherry-*URA3* | This study |
| AJY1158 | *sei1opi1Δ-PEX30*-2xmCherry | BY474? *MAT? his3Δ1 leu2Δ0 met15Δ0 ura3Δ0 sei1::KanMx6 opi1::HIS3 PEX30*-2xmCherry-*URA3* | This study |
| AJY1078 | *cho2Δ*-*PEX30*-2xmCherry | BY474? *MAT? his3Δ1 leu2Δ0 met15Δ0 ura3Δ0 cho2::HIS3 PEX30*-2xmCherry-*URA3* | This study |
| AJY1054 | *sei1cho2Δ* -*PEX30*-2xmCherry | BY474? *MAT? his3Δ1 leu2Δ0 met15Δ0 ura3Δ0 sei1::KanMx6 cho2::HIS3 PEX30*-2xmCherry-*URA3* | This study |
| AJY1016 | *opi3Δ*-*PEX30*-2xmCherry | BY474? *MAT? his3Δ1 leu2Δ0 met15Δ0 ura3Δ0 opi3::HIS3 PEX30*-2xmCherry-*URA3* | This study |
| AJY1021 | *sei1opi3Δ*-*PEX30*-2xmCherry | BY474? *MAT? his3Δ1 leu2Δ0 met15Δ0 ura3Δ0 sei1::KanMx6 opi3::HIS3 PEX30*-2xmCherry-*URA3* | This study |
| AJY1200 | *sei1pex30Δ*-*OPI1-*mCherry | BY474? *MAT? his3Δ1 leu2Δ0 met15Δ0 ura3Δ0 sei1::KanMx6 pex30::KanMx6 OPI1-*mCherry-*HIS3* | This study |
| AJY1055 | *ino1Δ-PEX30*-2xmCherry | BY474? *MAT? his3Δ1 leu2Δ0 met15Δ0 ura3Δ0 ino1::HIS3 PEX30*-2xmCherry-*URA3* | This study |
| AJY1053 | *sei1ino1Δ-PEX30*-2xmCherry | BY474? *MAT? his3Δ1 leu2Δ0 met15Δ0 ura3Δ0 sei1::KanMx6 ino1::HIS3 PEX30*-2xmCherry-*URA3* | This study |
| AJY1165 | *psd1psd2sei1Δ*-*PEX30*-2xmCherry | BY474? *MATa* *his3Δ1 leu2Δ0 met15Δ0 ura3Δ0 psd1::KanMx6 psd2::KanMx6 sei1::HIS3 PEX30-*2xmCherry-*URA3* | This study |
| AJY1060 | *sei1Δ*-*OPI1*-GFP-*PEX30*-2xmCherry | BY474? *MAT?* *his3Δ1 leu2Δ0 met15Δ0 ura3Δ0*  *sei1::KanMx6 OPI1-*GFP*-HIS3 PEX30-*2xmCherry-*URA3* | This study |
| AJY1199 | *are1are2dga1sei1Δ*-*GAL1-LRO1-PEX30-*2xmCherry | BY474? *MAT? his3Δ1 leu2Δ0 met15Δ0 ura3Δ0*  *are1::KanMx6 are2::KanMx6 trp1::URA3 sei1::HIS3 dga1::Lox-HIS-Lox TRP1-GAL1-LRO1 PEX30-*2xmCherry-*URA3* | This study |
| AJY1056 | *spo14Δ*-*PEX30*-2xmCherry | BY474? *MAT? his3Δ1 leu2Δ0 met15Δ0 ura3Δ0*  *spo14*::*HIS3 PEX30*-2xmCherry-*URA3* | This study |
| AJY1059 | *sei1spo14Δ*-*PEX30*-2xmCherry | BY474? *MAT? his3Δ1 leu2Δ0 met15Δ0 ura3Δ0*  *spo14*::*HIS3* *sei1::KanMx6 PEX30*-2xmCherry-*URA3* | This study |
