# Supplementary material for: Phosphatidic acid drives spatiotemporal distribution of Pex30 at ER-LD contact sites
Source: J Cell Biol. 2025 May 23;224(7):e202405162. doi: 10.1083/jcb.202405162 (PMC12101077; doi:10.1083/jcb.202405162)
Supplement: Table S2 — shows the plasmid list. [file jcb_202405162_tables2.docx]

**Table 2: Plasmid list**

| **Plasmid number** | **Name** | **Description** | **Source** |
| --- | --- | --- | --- |
| pAJ1140 | Ycplac111-*SEC63*-GFP | GFP fused to C-terminus of *SEC63* in Ycplac111 backbone; Leu2 and ampicillin selection markers | Laboratory collection |
| pAJ1144 | Ycplac111-*OPI1*-GFP | GFP fused to C-terminus of *OPI*1 in Ycplac111 backbone; used as PA sensor; Leu2 and ampicillin selection markers | This study |
| pAJ1006 | Yeplac181-*PEX30-*GFP | GFP fused to C-terminus of *PEX30* in Yeplac181 backbone; Leu2 and ampicillin selection markers | Laboratory collection |
| pAJ1193 | Yeplac181- *PEX30*(RHDΔ)-GFP | GFP fused to C-terminus of RHD(80-220 amino acids) truncated *PEX30* in Yeplac181 backbone; Leu2 and ampicillin selection markers | This study |
| pAJ1164 | Yeplac181-*PEX30*(DysFΔ)-GFP | GFP fused to C-terminus of DysF(284-408 amino acids) truncated *PEX30* in Yeplac181 backbone; Leu2 and ampicillin selection markers | This study |
| pAJ1194 | Yeplac181-*PEX30*(DUFΔ)-GFP | GFP fused to C-terminus of DUF(415-513 amino acids) truncated *PEX30* (415-513Δ) in Yeplac181 backbone; Leu2 and ampicillin selection markers | This study |
| pAJ1161 | pET15b-6xHis-DysF(280-410) | 6x-HIS tag fused to N-terminus of *PEX30* dysferlin domain | This study |
| pAJ1165 | pRS415-FYVE-GFP | GFP fused to C-terminus of FYVE domain; used as PI_3_P sensor; Leu2 and ampicillin selection markers | Will Prinz |
| pAJ1103 | Ycplac111-*OPI3* | *OPI3* ORF expressed in Ycplac111 backbone; Leu2 and ampicillin selection markers | This study |
| pAJ1216 | Yeplac181- *PEX30*(296-315Δ)-GFP | GFP fused to C-terminus of *PEX30* with dysferlin domain deletions in Yeplac181 backbone; Leu2 and ampicillin selection markers | This study |
| pAJ1218 | Yeplac181- *PEX30*(378-398Δ)-GFP | GFP fused to C-terminus of *PEX30* with dysferlin domain deletions in Yeplac181 backbone; Leu2 and ampicillin selection markers | This study |
