# Supplementary material for: Phosphatidic acid drives spatiotemporal distribution of Pex30 at ER-LD contact sites
Source: J Cell Biol. 2025 May 23;224(7):e202405162. doi: 10.1083/jcb.202405162 (PMC12101077; doi:10.1083/jcb.202405162)
Supplement: Table S3 — shows the primer list. [file jcb_202405162_tables3.docx]

**Table 3: Primer list**

| **Primer** | **Sequence** |
| --- | --- |
| RTN1 C' tag pFA6 F | TACAAAAAACTTGCAAAATGAATTGGAAAAAAACAACGCTcggatccccgggttaattaa |
| RTN1 C' tag pFA6 R | TTAGCTATTCTTGTTTGAAATGAAAAAAAAAAAGCACTCAgaattcgagctcgtttaaac |
| OPI1 KO pFA6 F | ATCAGGACAGTGTTTTTAACGAAGATACTAGTCATTGATGcggatccccgggttaattaa |
| OPI1 KO pFA6 R | CTGGTGGTAATGCATGAAAGACCTCAATCTGTCTCGGTTAgaattcgagctcgtttaaac |
| CHO2 KO pFA6 F | GATTTTCTTAGTGACAAAGCTTTTTCTTCATCTGTAGATGcggatccccgggttaattaa |
| CHO2 KO pFA6 R | AGTACTTTTTAAATATATATACTCAAAAAAAAAAAACTCAgaattcgagctcgtttaaac |
| OPI3 KO pFA6 F | AGCAATTGAAGACAACAAGAATAGCGCAAGTCAAGCGATGcggatccccgggttaattaa |
| OPI3 KO pFA6 R | GGCTTCTAACATTATAGAATATATAGAAATAGAGCACTTAgaattcgagctcgtttaaac |
| OPI1-mcherry-F5 | TTACGTAAAGCCCTCTCAGGACAACGTGGATAGCAAGGACggtgacggtgctggttta |
| OPI1-mcherry-R3 | CTGGTGGTAATGCATGAAAGACCTCAATCTGTCTCGGTTAtcgatgaattcgagctcg |
| INO1 KO pFA6 F | TTCATTCCCTTTTTTTTCCAGTGAAAAAGAAGTAACAATGcggatccccgggttaattaa |
| INO1 KO pFA6 R | TAGGCGGAAAAAGAAAAAGAGAGTCGTTGAAATGAGATTAgaattcgagctcgtttaaac |
| SEI1 KO pFA6 F | AAGGTTTCAAGAAAATAAGATAAAGTGAATAGGAAGGATGcggatccccgggttaattaa |
| SEI1 KO pFA6 R | TAGGTTTTAAAATTATATAGCGAGAAGTACAATTCTATCAgaattcgagctcgtttaaac |
| OPI1 C' tag pFA6 F | TTACGTAAAGCCCTCTCAGGACAACGTGGATAGCAAGGACcggatccccgggttaattaa |
| OPI1 C' tag pFA6 R | CTGGTGGTAATGCATGAAAGACCTCAATCTGTCTCGGTTAgaattcgagctcgtttaaac |
| SPO14 KO pFA6 F | ACGCAAGAAGAAAAGGTAGGATAGATAAACAAGGGTGATGcggatccccgggttaattaa |
| SPO14 KO pFA6 R | CAGGTAATGGTGTGTTCCTGGTCGTTTTTATATTCCCTTAgaattcgagctcgtttaaac |
| Yep181-Opi1-500 | ggataacaatttcacacaggaaacagctatgaccatgattTACTGTACATGCAGTGGCCT |
| Yep181-tADH1-R | gttttcccagtcacgacgttgtaaaacgacggccagtTATTACCCTGTTATCCCTAGCGG |
| Pex30 284-408 deletion-R | TCATCAAAGTCAGAAGTTTTAGGCTTGGAATCGGATAATA |
| Pex30 284-408 deletion-F | TATTATCCGATTCCAAGCCTAAAACTTCTGACTTTGATGA |
| Pex30 415-513 deletion-R | TTGCTATCGCGACCAATGGTTTCATCAAAGTCAGAAGTTTTGACC |
| Pex30 415-513 deletion-F | GGTCAAAACTTCTGACTTTGATGAAACCATTGGTCGCGATAGCAA |
| Pex30 80-220 deletion-R | GCTACTTTAGACCATGGTGAAGGGTACAATTTCACCAACG |
| Pex30 80-220 deletion-F | CGTTGGTGAAATTGTACCCTTCACCATGGTCTAAAGTAGC |
| His-DysF(280-410)BamH1F | CAGCCATATGCTCGAGGATCCATCTAGAATGGATTCCAAGCCTATCCGTTT |
| His-DysF(280-410)BamH1R | GCTTTGTTAGCAGCCGGATCCTTAGTCGACTCAAGTTTTGACCAATTCTGCGG |
| Yep181-Opi3 F -500 | ggataacaatttcacacaggaaacagctatgaccatgattAACTCCGTCAGGTCTTCCAC |
| Yep181-Opi3 R +300 | agggttttcccagtcacgacgttgtaaaacgacggccagtATTACGTATGGTGGCGTTTT |
| DysF MC1 R | TCATTCAAAAATTCATCGGTCTGGTTCTCGTACAAAACA |
| DysF MC1 F | ATGTTTTGTACGAGAACCAGACCGATGAATTTTTGAATGA |
| DysF MC2 R | GCGGTTCTTACCCATCTTCTTATAAATCCCTCGTCAGGGG |
| DysF MC2 F | CCCCTGACGAGGGATTTATAAGAAGATGGGTAAGAACCGC |
