# Supplementary figures and images for: Phosphatidic acid drives spatiotemporal distribution of Pex30 at ER-LD contact sites
Source: J Cell Biol. 2025 May 23;224(7):e202405162. doi: 10.1083/jcb.202405162 (PMC12101077; doi:10.1083/jcb.202405162)

Figure 3F

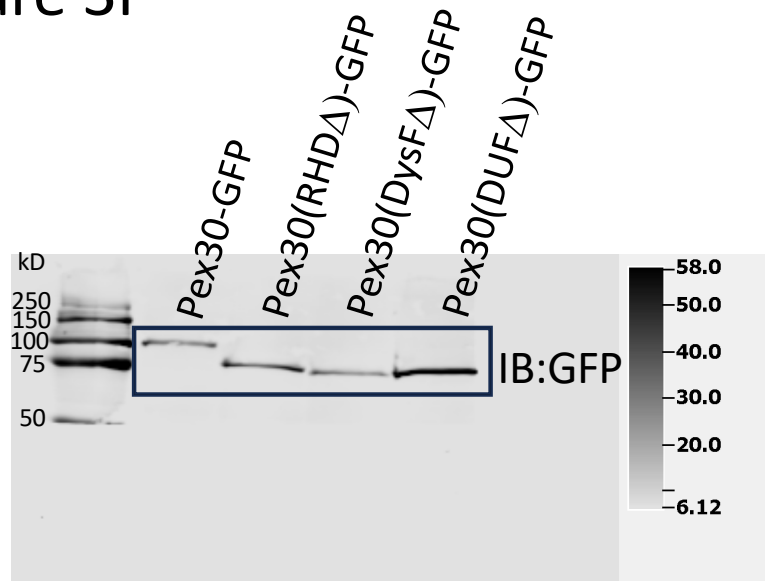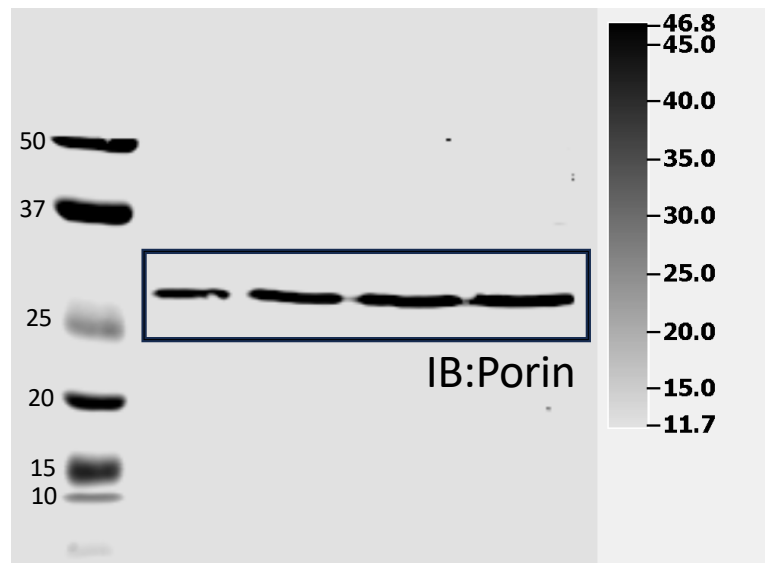

Supplement: SourceData F3 — is the source file for Fig. 3. [file jcb_202405162_sourcedataf3.pdf]

Figure 4

**B**

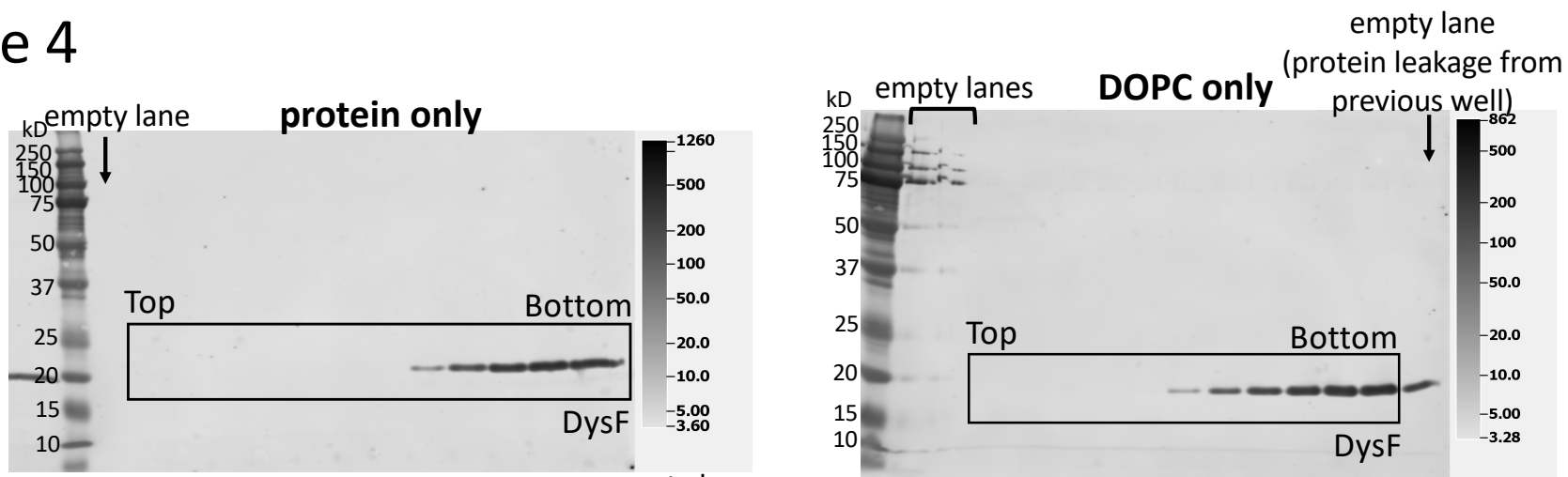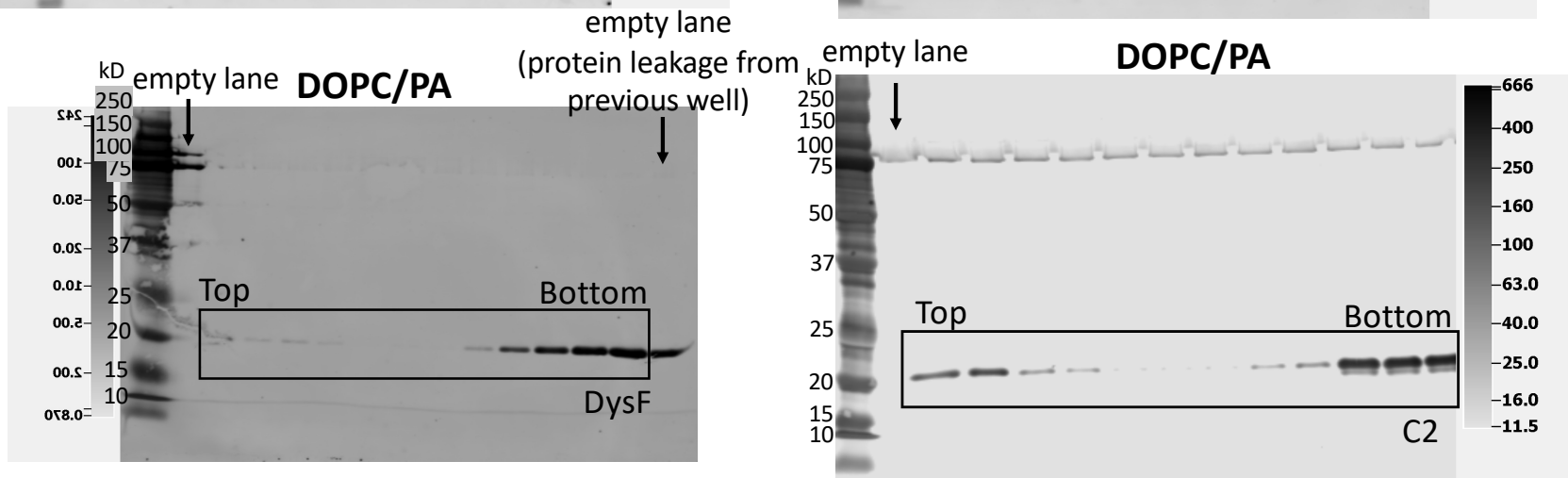

**C**

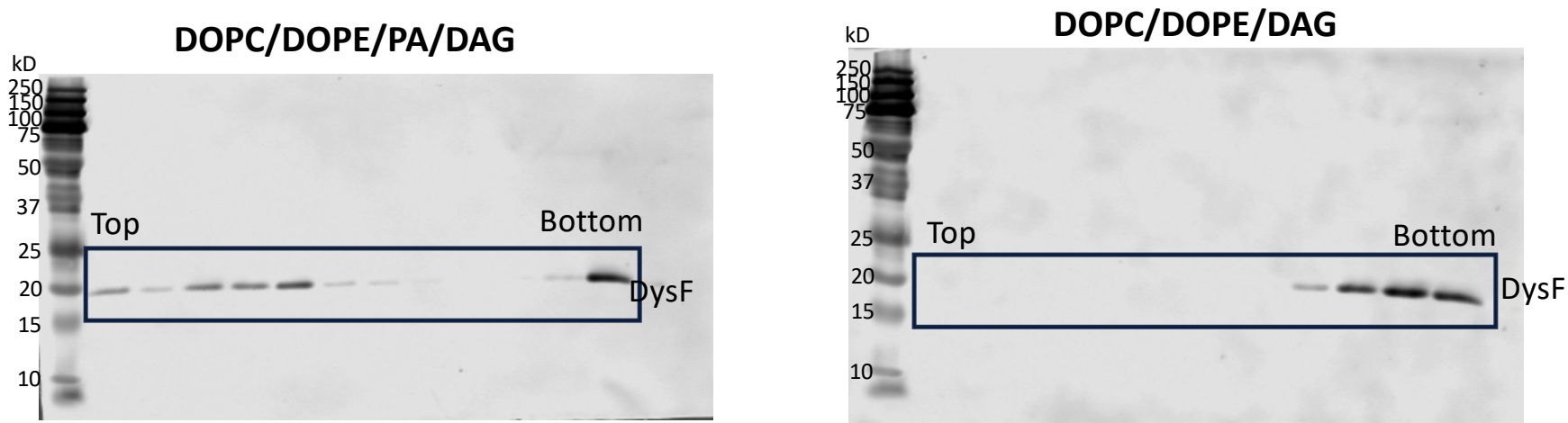

Supplement: SourceData F4 — is the source file for Fig. 4. [file jcb_202405162_sourcedataf4.pdf]

Figure 5G

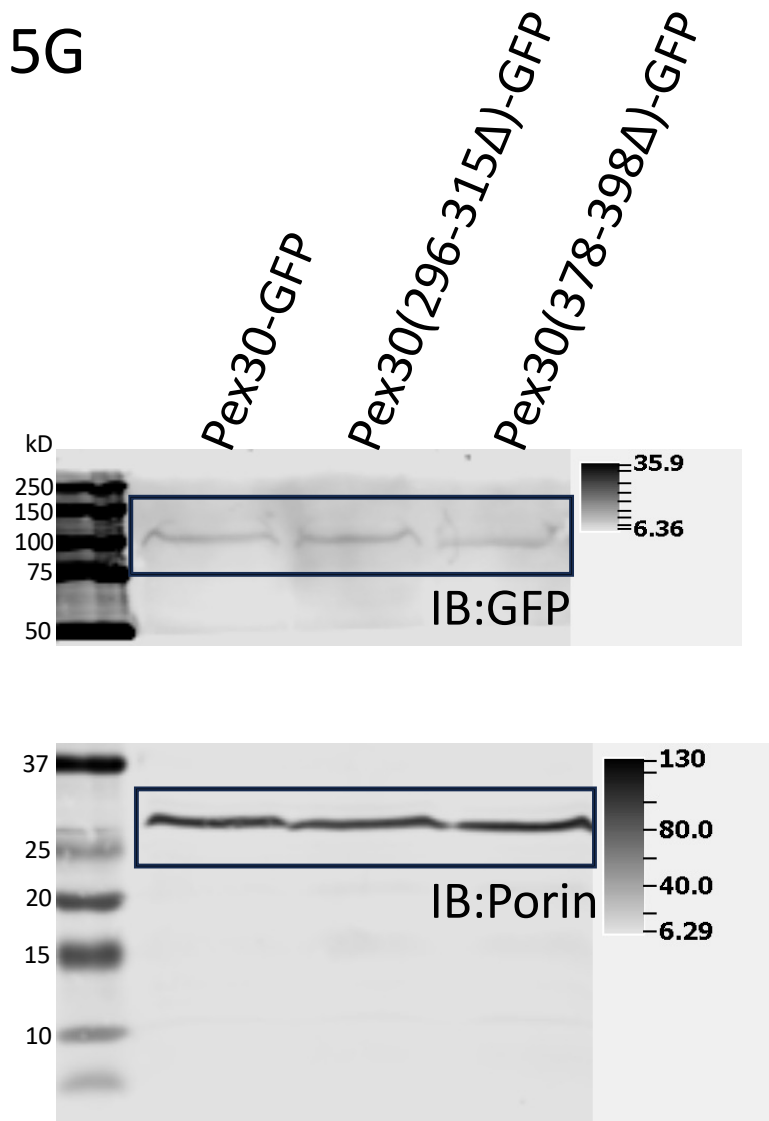

Supplement: SourceData F5 — is the source file for Fig. 5. [file jcb_202405162_sourcedataf5.pdf]

# Supplemental Figure 1A

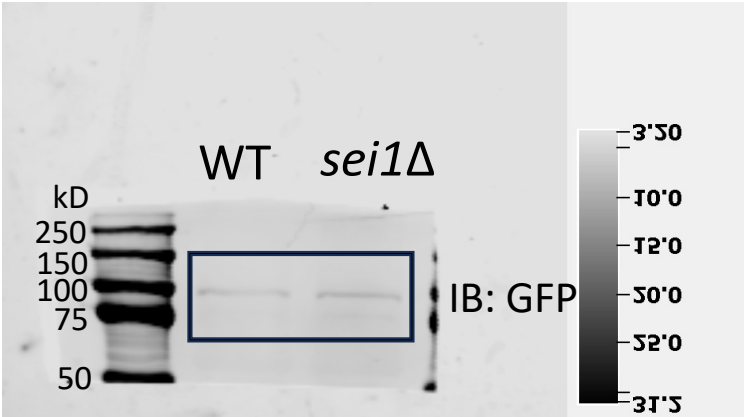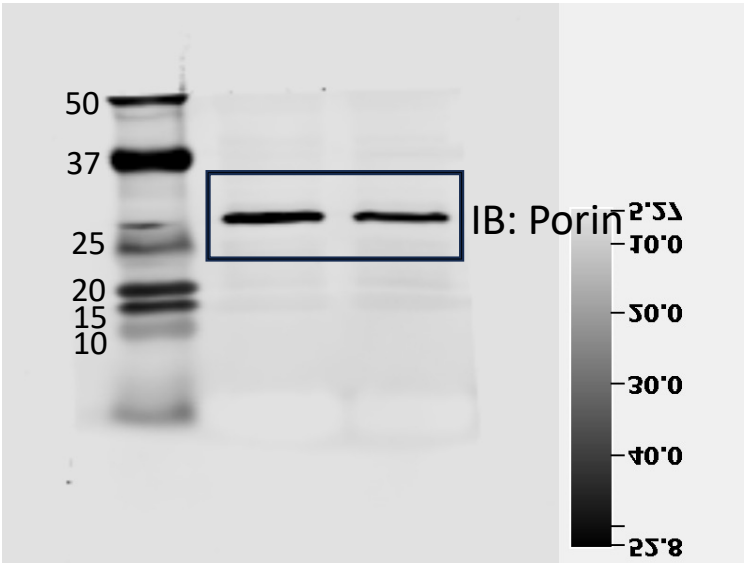

Supplement: SourceData FS1 — is the source file for Fig. S1. [file jcb_202405162_sourcedatafs1.pdf]

# Supplemental Figure 2

**A**

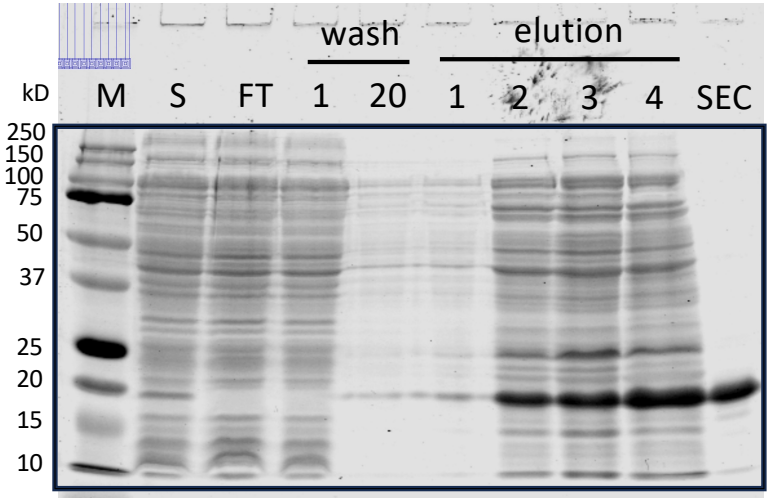

**C**

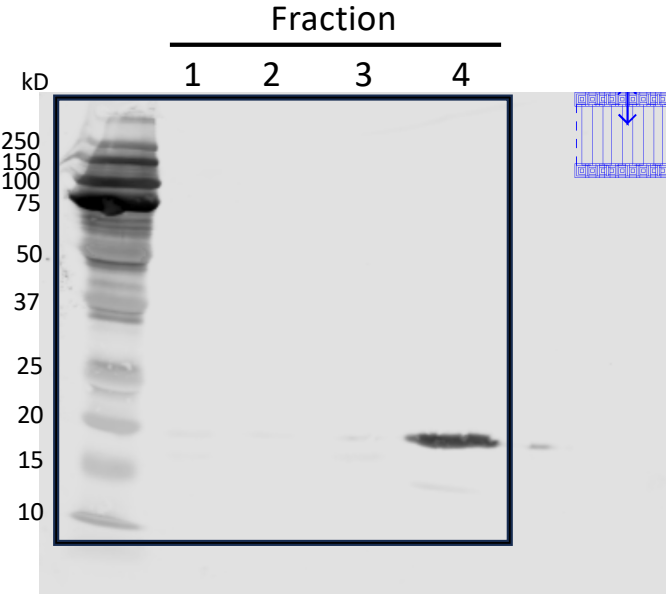

**F**

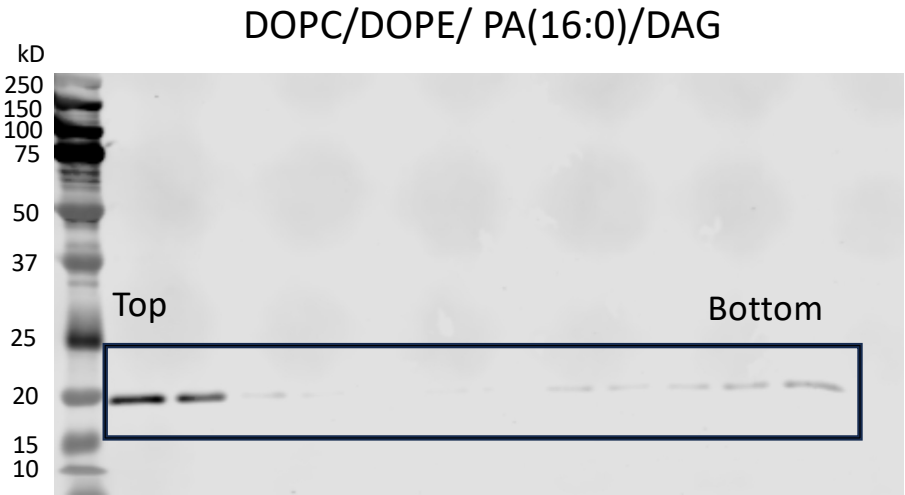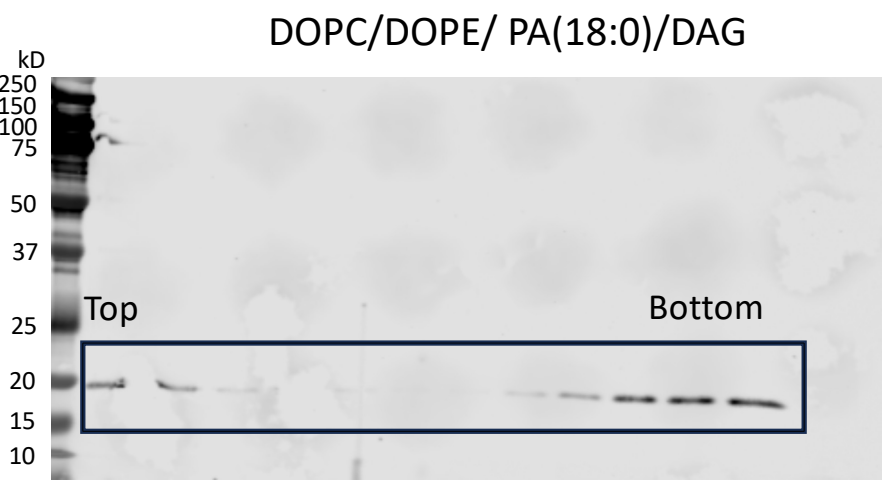

Supplement: SourceData FS2 — is the source file for Fig. S2. [file jcb_202405162_sourcedatafs2.pdf]
